# A Review on Photoacoustic Spectroscopy Techniques for Gas Sensing

**DOI:** 10.3390/s24206577

**Published:** 2024-10-12

**Authors:** Dakshith Ruvin Wijesinghe, Md Abu Zobair, Mina Esmaeelpour

**Affiliations:** 1 Mining and Explosive Engineering Department, Missouri University of Science and Technology, Rolla, MO 65401, USA; 2 Electrical and Computer Engineering Department, Missouri University of Science and Technology, Rolla, MO 65401, USA

**Keywords:** photoacoustic spectroscopy, acoustic cells, all-optical acoustic detectors, trace gas

## Abstract

The rapid growth of industry and the global drive for modernization have led to an increase in gas emissions, which present significant environmental and health risks. As a result, there is a growing need for precise and sensitive gas-monitoring technologies. This review delves into the progress made regarding photoacoustic gas sensors, with a specific focus on the vital components of acoustic cells and acoustic detectors. This review highlights photoacoustic spectroscopy (PAS) as an optical detection technique, lauding its high sensitivity, selectivity, and capability to detect a wide range of gaseous species. The principles of photoacoustic gas sensors are outlined, emphasizing the use of modulated light absorption to generate heat and subsequently detect gas pressure as acoustic pressure. Additionally, this review provides an overview of recent advancements in photoacoustic gas sensor components while also discussing the applications, challenges, and limitations of these sensors. It also includes a comparative analysis of photoacoustic gas sensors and other types of gas sensors, along with potential future research directions and opportunities. The main aim of this review is to advance the understanding and development of photoacoustic gas detection technology.

## 1. Introduction

The push for modernization and industrialization on a global scale has resulted in increased emissions from various gas sources, raising significant environmental and health concerns. This has spurred the pursuit of alternative energy sources and the need to reduce greenhouse gas emissions to promote a low-carbon economy. Consequently, there is a growing demand for precise, sensitive, and discerning gas-monitoring technologies to measure gas concentrations and evaluate air quality [1,2] in environmental monitoring, industrial process optimization, food quality control, medical pre-screening, and other areas [3,4,5]. The potential impact of this research on the global issue of gas emissions is significant. It is crucial to detect hazardous gases such as nitrogen oxides, carbon monoxide, hydrogen sulfide, and methane to minimize pollution and protect human health [6]. Numerous research efforts have been dedicated to designing innovative gas sensors, and recent developments in monitoring and detecting gas leaks are mainly categorized into contact-type gas sensors and non-contact gas detectors [7]. Contact-type gas sensors use specific sensing elements or material properties that change when exposed to the target gas. These types of sensors include electrochemical sensors [8], mechanical sensors, acoustic sensors [9], triboelectric sensors [10], thermoelectric sensors [11], and work-function-based sensors [12]. However, many of these sensors have limitations, such as sensitivity thresholds (ppm) and cross-sensitivity to other gases, sensitivity to environmental conditions, performance degradation over time, and the need for frequent recalibration [13].

In response to these challenges, the pursuit of ultra-sensitive, highly selective, repeatable, and non-contact gas monitoring has driven the development of various optical detection techniques based on optical phenomena such as infrared absorption, Raman scattering, and the photoacoustic effect [14]. These sensing techniques include Raman spectroscopy [15], Raman Lidars [16], photoacoustic spectroscopy (PAS) [17], and the use of non-dispersive infrared (NDIR) gas sensors [18]. Other optical techniques such as noise-immune cavity-enhanced optical heterodyne molecular spectroscopy (NICE-OHMS) [19], cavity ring-down spectroscopy (CRDS) [20], dual-comb optical spectroscopy [21], and tunable diode laser absorption spectroscopy (TDLAS) [22,23] have also been developed and utilized for gas sensing. While some of these techniques, such as CRDS and NICE-OHMS, can offer extremely high sensitivity, they often come at the cost of system complexity and high costs. Among these techniques, TDLAS is simpler and more widely used, but generally cannot achieve the sensitivity of the more advanced techniques. Raman Lidars, on the other hand, can be useful for remote gas-sensing applications, but they also suffer from low sensitivity. Nevertheless, due to its potential for miniaturization, high selectivity, repeatability, low costs, and high sensitivity PAS stands out as one of the most promising techniques for gas sensing. In this technique, the photoacoustic effect is exploited to measure gas concentrations to enable the detection of a wide range of gaseous species [14].

Hence, this literature review examines the latest advancements in photoacoustic gas detection, paying special attention to crucial elements such as acoustic cells and acoustic detectors, which play significant roles in enhancing the discussed sensors’ overall effectiveness. This paper is structured as follows: First, it outlines the principles of photoacoustic gas sensors in Section 2, followed by an overview of recent developments in photoacoustic gas sensor components in Section 3. The application of photoacoustic gas sensors is briefly discussed in Section 4, along with the associated challenges and limitations in Section 5. Future directions and opportunities are explored in Section 6. Finally, Section 7 offers the conclusion.

## 2. Principles of PAS Gas Sensors

Most PAS sensors consist of four primary components: an acoustic sensor or microphone, a photoacoustic (PA) cell, a light source, and a signal-processing unit. The PAS technique utilizes modulated light absorbed by the gas molecules. This process generates heat, which in turn is converted into gas pressure through the non-radiative relaxation of excited molecules. An acoustic sensor then detects the resulting gas pressure gradients as modulated acoustic pressure, indicating the quantity or concentration of gas molecules present [24]. The absorption wavelength of the modulated light is chosen based on the type of gas being detected [25], and the laser modulation frequency is dictated by the acoustic sensor’s frequency response [26]. Some sensors also include an optical cavity in conjunction with the acoustic cell to improve optical power and maximize acoustic pressure [27]. Modulation of the light source and/or the dimensions of the PA cell can be precisely regulated to ensure effective interaction with gas molecules, leading to the generation of acoustic waves at resonant frequencies for accurate gas identification [28].

The PA cell is usually a sealed gas chamber with gas and optical inlet and outlet ports and can be classified as non-resonant or resonant based on how it operates [24]. Resonant cells utilize acoustic resonances for gas detection, enhancing the acoustic pressure and making them suitable for detecting trace gases [29]. In contrast, non-resonant cells operate using a much lower modulation frequency than the resonant frequency, usually within the range of a few tens of Hz [30]. Different methods, such as density-based spatial clustering and principal component analysis, make use of the non-resonant cell for distinguishing and classifying multiple gas components [31].

In the PA cell, the absorption of the laser light by the gas molecules induces heat production and the generation of acoustic waves. The amplitude of the heat production rate is expressed as $H_{0}\left(W/m^{3}\right)=\alpha CI$ [32], where C is the concentration of the trace gas, and $I=I_{0}\exp\left(-\alpha l\right),$ according to the Beer–Lambert absorption law [33]. In this law, I represents the transmitted light intensity, $I_{0}$ is the incident light intensity, *α* denotes the absorption coefficient of the gas, and *l* stands for the absorption path length. The propagation of the resulted acoustic pressure (p) in the PA cell can be determined using the standard Helmholtz equation [34]:

(1) $$\nabla^{2}p-\frac{1}{v_{a}^{2}}\frac{\partial^{2}p}{\partial t^{2}}=-\frac{\left(\gamma_{sp}-1\right)}{v_{a}^{2}}\frac{\partial H}{\partial t}$$

where $v_{a}$ is the velocity of sound, and $\gamma_{sp}$ is the ratio of the specific heat of the gas at constant pressure $C_{p}$ to its specific heat at constant volume $C_{v}$. This equation can be analytically solved for simple structures such as a cylindrical cell [35]. Additionally, a lumped element model [30] can be utilized to describe the acoustic behavior by using the analogy theory of the one-dimensional electric transmission line [36,37,38]. The lumped acoustic model is a simplified approach used to describe acoustic systems where the wavelength of the sound is comparable to the physical dimensions of the components. In this model, the acoustic elements (such as cavities, tubes, or diaphragms) are treated as lumped elements, meaning that their properties like mass, compliance, and resistance are assumed to be concentrated at specific points rather than distributed over space.

Equation (1) does not include the effects of acoustic loss produced by heat conduction and viscosity, and to properly discuss the PA effect, it is necessary to include these mechanisms. Although this can be achieved by modifying Equation (1), the resulting equation and its solution are unnecessarily complex. Instead, the Navier–Stokes model [39] provides an accurate description of the thermo-viscous acoustics problems, which can be solved through the finite element analysis (FEM) method, and simulation tools like COMSOL Multiphysics can be utilized. This model is described by the following set of equations, known as the continuity equation, the momentum equation (the Navier–Stokes equation), and the energy equation, respectively [40]:

(2) $$\frac{d\rho }{dt}+\rho \left(\nabla \cdot v\right)=0\;$$

(3) $$\rho \frac{dv}{dt}=\nabla \cdot \sigma +F\;$$

(4) $$\rho C_{p}\frac{dT}{dt}-\alpha_{p}T\frac{dp}{dt}=-\nabla \cdot q+H$$

The divergence of the velocity field, v, is related to the variation of density, ρ, in the continuity equation, Equation (2). The momentum equation, Equation (3), connects the fluid acceleration, dv/dt, to a general volumetric force, F, as well as forces coming from the viscosity tensor, σ. Lastly, the temperature and pressure fluctuations are connected to an energy source term, H, and the energy lost through conduction, whose heat flow is given by q=−κ∇T, via the thermal diffusion equation, also known as the energy equation, Equation (4). Here, $C_{p}$ is the heat capacity at constant pressure, κ is the thermal conductivity, and $\alpha_{p}$ is the coefficient of thermal expansion (isobaric) of the gas that acts as the fluid medium. These equations can be solved using the thermo-viscous acoustics module in COMSOL Multiphysics.

To compare different systems with varying influencing factors, a generalized standard equation such as normalized noise equivalent absorption (NNEA) is essential as it is independent of the gas types. The NNEA is expressed as [24]

(5) $$NNEA=\frac{\alpha_{min}P}{\sqrt{\Delta f}}\;\left[W{cm}^{-1}{Hz}^{-1/2}\right]$$

where $\alpha_{min}$ represents the minimum detectable absorption coefficient, *P* denotes the optical power, and Δf is the equivalent noise detection bandwidth of the acoustic detector. Also, another figure of merit is the minimum detection limit (MDL), sometimes also referred to as the minimum detectable concentration or noise-equivalent concentration, which is the lowest concentration of gas that can be measured under any given experimental condition and using any measurement technique. Typically, this unit is in parts per million (ppm), parts per billion (ppb), or parts per trillion (ppt).

## 3. Recent Advances in PAS Gas Sensors

Photoacoustic spectroscopy gas sensors are increasingly selected over other gas sensing techniques due to their sensitivity, size, repeatability, and response time, to name a few advantages, leading to significant advancements in this field. Specifically, progress is noticeable regarding acoustic cells, sensor development, signal processing, and machine learning. These developments have improved sensitivity, accuracy, and speed; reduced maintenance and operational costs; and minimized the impact of environmental factors. As a result, we examined the recent advancement in these areas.

### 3.1. Acoustic Cell

As mentioned earlier, there are primarily two types of acoustic cells: non-resonant and resonant. Non-resonant cells do not harness the resonance effect of acoustic waves in the cell structure and only take into account the direct impact of sound waves generated through the PAS effect on the microphone. In contrast, resonant cells produce acoustic resonance at specific frequencies, which are the eigenmodes of the acoustic cell structure. These eigenmode profiles and frequencies can be calculated from the acoustic wave equation for the cell structure along with the boundary conditions. The resonance effect helps to improve the acoustic pressure generated through the PAS effect and is especially important when the gas concentration or absorption coefficient is low.

Operating the PAS sensor at resonance necessitates matching the resonance frequency of the PA cell and the acoustic sensor (microphone) with the laser modulation frequency. The excitation laser can be intensity and/or wavelength modulated. Intensity modulation (IM) can be accomplished by employing an optical chopper in the optical path of the laser or by modulating the laser current with a high-frequency signal. In wavelength modulation (WM), the laser is modulated around the center of the gas absorption line. A high-frequency sinusoid is superimposed on a low-frequency ramp signal/sinusoid (few Hz), and this doubly modulated current waveform is directly injected into the laser current controller. The low-frequency component slowly sweeps the wavelength across the gas absorption line, while the high-frequency component introduces additional WM as the IM arising from the high-frequency component varies the refractive index of the gain medium of the laser. This results in simultaneous and synchronous WM and IM that have a phase difference. The detection process involves the recovery of signals at various harmonics of the modulation frequency (generally the first two harmonics, 1f and 2f), which can be achieved using a lock-in-amplifier. In 1f detection, the acquired signal makes a significant contribution arising from the direct IM of the laser, which is why the 2f signal is most widely used to recover the wavelength-modulated signal. More details regarding wavelength modulation spectroscopy can be found in [41,42].

While feedback loops can keep the sensor operating at the resonance point, they affect the sensor’s real-time response. As a solution, optical resonance systems can be utilized to increase the laser power in the gas cell as well as the acoustic pressure by increasing the optical path. Additionally, improving the design of the acoustic cell has been identified as a method for enhancing the efficiency of PAS gas sensors, and there have been notable advancements in this area over the past few years. As a result, we have examined the progress made in acoustic cell design technology.

**Figure 1:**
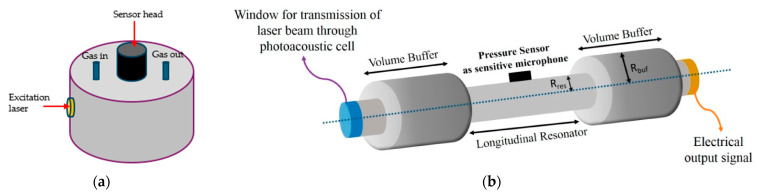
Schematic diagram of two typical cylindrical gas cells: (**a**) an aluminum cylindrical (disk) cell with an excitation laser on the side—whose presence results in an increased optical pathway due to multiple reflections—with an acoustic sensor placed at the top of the cylindrical structure and (**b**) a cylindrical cell with two buffer regions [43] (known as an H or dumbbell cell) usually used for resonant PAS detection. Mirrors can be used in the buffer regions to increase the optical pathway.

Cell geometry plays an important role in the PAS technique. Cylindrical cells and their different variations are the most commonly used cell structures for PAS gas detection. They naturally support standing acoustic waves, especially in the radial and longitudinal directions. This property enhances the strength of the generated acoustic signals. By carefully designing the cell’s dimensions, its acoustic resonance frequencies can be tuned to match the resonance of the acoustic sensor and the modulation frequency of the excitation laser. This resonance amplifies the pressure waves created by the PA effect, leading to stronger and more detectable acoustic signals. A cylindrical geometry also helps create a more uniform pressure distribution inside the cell. This is important for producing strong, uniform acoustic signals that are easier to detect and analyze. It also allows the strategic placement of the acoustic sensors at locations where the acoustic pressure is strongest (e.g., near the antinodes of standing waves), which maximizes the efficiency of sound wave detection. Furthermore, cylindrical resonators are easier to fabricate and tune to specific resonance frequencies, making it possible to enhance sensitivity. Figure 1 shows two typical cylindrical gas cells used for PAS gas sensors. The difference between the two systems mainly lies in the placement of the acoustic sensor and excitation laser as well as the operation mode of the sensor. One is a disk with a radius larger than its length, and the other is a cylinder with a length larger than its radius which is encapsulated by two buffer regions at both ends.

While it is simple to design closed cylindrical cells and integrate them into a PAS detection system, they limit acoustic signal detection by introducing noise. This noise is mainly produced by absorption by the laser windows and turbulence at the gas inlet and outlet. A solution to this problem is using an open-ended cylinder. A common form of open-ended cylinder used in the PAS technique is the H-shaped cell shown in Figure 1b. The mentioned figure was modified to show an open-ended cylindrical tube forming the acoustic resonator with buffer volumes at each end. The shortest length of the buffers that minimizes coupling of the background noise with the resonator is a quarter of a wavelength [44]. In this structure, the buffer is placed at the end of the resonator and connected to gas inlet and outlet valves, and the resulting PA signals are collected using a highly sensitive microphone. For micro-resonator tubes, generally with diameters of the order of a few millimeters, an end correction is applied to the cell length considering sound radiation into the buffer regions at the ends due to the mismatch between the one-dimensional acoustic field inside the pipe and the three-dimensional field outside radiated by the open end [45,46,47,48,49]. The correction to the cell length is Δl=16r/3π, where r is the cell diameter between the two buffers. This changes the resonance frequency of the cell, which ultimately influences the PA signal and background signal. Several experiments were conducted to study the effects of the buffer and understand how the cell’s geometry affects the internal acoustic pressure [44,50].

In addition to conventional cell configurations such as the cylindrical and H-shape cells, various other cell arrangements have been designed, demonstrated, and utilized for PAS gas sensors over the past decades to improve both sensitivity and noise reduction. For instance, differential PA cells consist of two identical acoustic resonators that are used to reduce noise and improve sensitivity; Helmholtz cells are made of two chambers connected by a small tube, a configuration resulting in higher sensitivity and better frequency-response shaping; and the differential Helmholtz PA cell (DHPAC) consists of two chambers connected by two small tubes, constituting a combination of the two techniques [51]. Instead of using buffer cylinders in H-shaped cells, Zhang et al. [52] employed box-shaped buffers and two mufflers at the inlet and outlet. Zhu et al. [53] developed a high-quality-factor spherical PA cell for acetylene (C_2_H_2_) detection with an MDL of 106.8 ppb at 1 s and 11.03 ppb at 100 s averaging time. Wu et al. [54] considered the Y shape in the resonator cell’s design and applied it to detect CH_4_, achieving an MDL of 52.8 ppb within 100 s averaging time. Furthermore, Wu et al. [55] coupled a sphere and a cylinder, developing a sphere–cylinder coupled acoustic resonator for high-sensitivity trace gas sensing, while Zhang et al. [56] developed an O-shaped multi-pass cell to detect trace C_2_H_2_. Moreover, Wang et al. [57] designed a novel trapezoid compound ellipsoid resonant PA cell (TCER-PAC).

#### Optimization

Cell Material—For the fabrication of the PA cell, the choice of fabrication material is important as it can affect the PAS sensor performance. For example, the cell conductivity allows for exhausting the generated temperature within the cell, while the reflection capability of the inner walls allows for increased absorption of energy into the gas. Hence, different techniques and fabrication materials have been used in acoustic cell designs. Chen et al. [58] examined a brass cavity cylinder with a volume of 848 µL and used an angular collimator laser to generate multiple reflections on the inner wall. This approach increased the length of the absorption path, leading to an MDL of 31 ppb for a trace gas in 400 s of averaging time. Mao et al. [59] designed and optimized the DHPAC such that it was endowed with a 37 mL capacity, and they installed concave mirrors on each side of one chamber to allow for multiple passes of the light source. This configuration effectively increased excitation and reduced noise interference, resulting in an impressive MDL of 6.6 ppb for CH_4_ in 100 s. Furthermore, Lima et al. [60] utilized 3D-printing technology along with photosensitive liquid resin to construct the acoustic cell.

Cell Geometry—Cell size and geometry are critical for practical applications. Therefore, multiple attempts have been made to optimize the acoustic cell’s performance while reducing its size through numerical optimization and experimental validation. Since the optimization process involves a combination of multiple dimensional variations, it is an expensive process. Hence, the numerical approach is more attractive for optimizing dimensions, and experiments are conducted on the final optimized design to validate it. As a result, numerous experiments have been conducted to optimize the size of the acoustic cell using numerical approaches. For instance, the optimization of the buffer structure [50] and the T-type acoustic cell dimensions [61] can be cited in this regard. Further, Bijnen et al. [62] optimized the acoustic cell numerically and experimentally evaluated the performance of the final design. In addition, numerical optimization techniques have been considered for the topological shape optimization of the acoustic cell [63,64].

Excitation Laser—The strongest molecular absorption lines typically correspond to the near-infrared and mid-infrared parts of the electromagnetic spectrum. The mid-infrared absorption lines are typically stronger than the near-infrared lines, which is why the characteristics of the light source are also important. Various types of light sources, such as laser diodes [65], UV lasers [66], tunable lasers [67], quantum cascade lasers [68], fiber optic lasers [69], and near-infrared lasers [70], are utilized in acoustic sensors based on factors like power consumption and complexity for gas excitation as heat sources to trigger the PA process. Therefore, numerical optimization techniques can and have been employed to optimize the necessary light source characteristics, sensor dimensions, sensor placement, and cell dimensions [44,71].

Acoustic sensor placement—When designing the acoustic cell, it is crucial to consider the placement of the acoustic sensor (microphone) and the light source [24]. In resonant acoustic cells, the detection sensor is typically placed at the highest acoustic pressure zone, and in non-resonant cells, the sensors are positioned opposite to the light source or alongside its path. While simple acoustic mode profiles have been theoretically calculated for cylindrical cell structures (with open and closed ends) using the acoustic wave equation, more complex structures require numerical simulations. Such simulations can be made with finite element solvers such as COMSOL by numerically solving the full linear Navier–Stokes equations [40].

Multi-gas detection—Currently, typical PAS gas sensors can only identify specific gases. The need for multiple sensors limits their ability to detect multiple gases simultaneously. To address this limitation, several alternative techniques have been developed. Primarily, multiple-gas detection is achieved by combining multiple acoustic cells into a single cell. Yin et al. [70] developed a dual-resonator-structure PA cell by combining two parallel cells with two lasers and two microphones to detect CO and H_2_S with 31.7 ppb and 342.7 ppb MDLs. Li et al. [72] combined three acoustic cells with different lengths that were connected to two microphones and three lasers to simultaneously detect C_2_H_2_, NO, and CF_4_ with MDLs of 480 ppb, 260 ppb, and 0.57 ppb, respectively. Xiao et al. [73] designed a double-channel differential T-shape cell with a first-order resonant frequency of 610 Hz, achieving an MDL of 36.45 ppb of CH_4_ in 1 s of averaging time_._ As an alternative for combining multiple acoustic cells, Huang et al. [74] considered a spherical cavity cell with different lasers along with a single sensor to detect H_2_O, CO_2_, and CH_4_. Additionally, several attempts have been made to develop a tunable laser source to modulate wavelengths with a single acoustic cell to detect multiple gases [67,75]. Furthermore, Er et al. [76] presented findings on a new multi-gas detection system featuring a uniquely designed cruciform PA cell (CF-PAC). The CF-PAC includes two beam paths and a single detection sensor and is designed to accommodate multiple independently incident laser beams originating from different directions, making it particularly useful for scenarios where it is challenging to combine beams due to significant wavelength variations.

### 3.2. Acoustic Sensor

The acoustic sensor is an important component of PAS gas sensors as it converts the acoustic waves into an electrical signal, which can be processed using signal-processing techniques. The main tools used for transduction are the electromechanical sensors and all-optical transducers discussed in the following sections.

#### 3.2.1. Electromechanical Sensors

These sensors convert the acoustic wave (or the pressure gradient) into an electrical signal through electro-mechanical conversion. Commercially available capacitive microphones fall into this category. The traditional transduction technique for PAS gas detection with a capacitive microphone can be traced back to the 1970s. It is made of two parallel plates with an air gap between them—a fixed backplate and a moveable top diaphragm [77,78,79]. When two plates are linked to different electrodes, parallel capacitance is produced. The diaphragm moves in response to the acoustic wave striking the condenser, altering the gap between the plates and generating a change in capacitance that can be measured using a special electrical circuit. Recently, capacitive microphones have benefited from miniaturization due to the advent of the microelectronic and microelectromechanical system (MEMS) industry [80]. Capacitive microphones based on MEMS are constructed from silicon wafers and incorporated into smaller devices. Standard MEMS capacitive microphones are affordable, highly sensitive, and have a small footprint. These microphones have the advantages of being compact and inexpensive, but they also exhibit thermal instability and a flat response over the audio frequency range, reducing sensitivity. Recently, a PAS sensor with piezoresistive MEMS transducers was developed by Lhermet et al. [81]; here, a variation in the electrical resistance occurs in response to mechanical stress. Employing the standard silicon microfabrication technique, the sensor’s dimensions were greatly reduced as the transducer was directly fabricated within the gas cell.

An alternative that is frequently used to boost sensor sensitivity is the quartz tuning fork (QTF) resonator, which gave rise to the quartz-enhanced PAS (QEPAS) approach. The QTF is a resonator composed of two quartz prongs that are joined at one end, and its shape and piezoelectricity determine its resonance frequency. This piezoelectricity is the result of the QTF material’s ability to generate electrical charges through the deformation of the crystal structure. Using a QTF resonator, the mechanical stress generated by the acoustic wave is transformed into an electrical signal. Large quality factors can be achieved by effectively containing the acoustic energy in the tuning fork’s prongs thanks to the rigidity of quartz.

**Figure 2:**
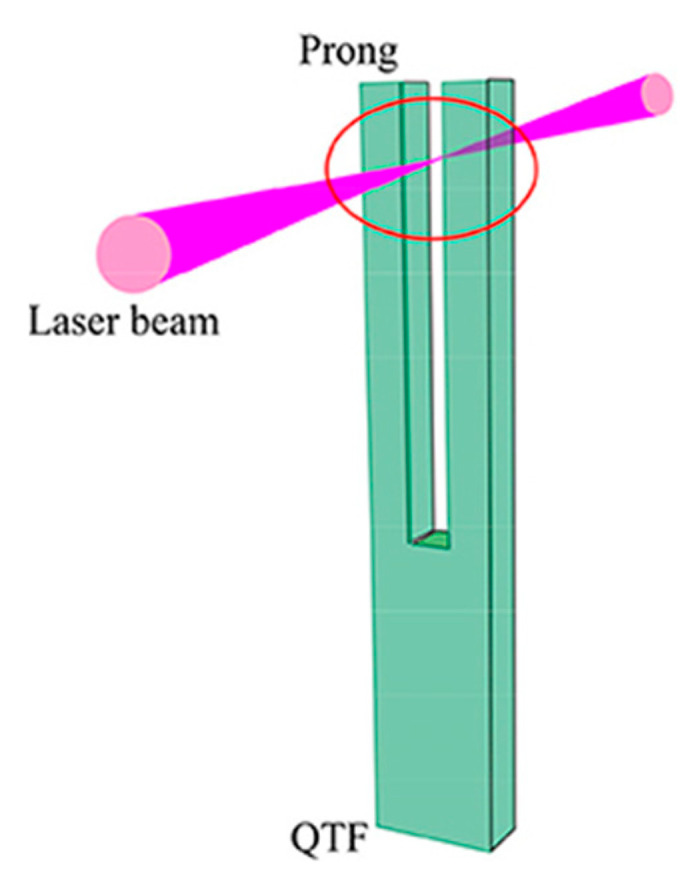
Schematic of QEPAS on-beam configuration [94].

The QEPAS technique was introduced for the first time by Kosterev et al. [82]. In this technique, the laser beam is modulated at the QTF resonance frequency as it is focused between the tuning fork prongs, as shown in Figure 2. Signal generation using the prongs of the QTF is enhanced by introducing acoustic resonators, including one or two thin tubes. The QTF is placed next to (off-beam QEPAS) or between (on-beam QEPAS) the tubes to measure the acoustic vibrations that are generated in the gas filling the tubes [83]. The sensitivity of the QEPAS system is significantly impacted by the size and positioning of the tiny pipe resonators. For on-beam QEPAS, an NNEA value as low as 2.7 × 10^−10^ W cm^−1^ Hz^−1/2^ has been reported [84,85,86,87], while for off-beam QEPAS, an NNEA value as low as 4.1 × 10^−9^ W cm^−1^ Hz^−1/2^ was reached [88,89,90,91]. Borri et al. [92] proposed a tuning fork with custom dimensions to lower its resonance frequency to 4.2 kHz, thus lying below the frequency limit set by the relaxation rate of the target gas, and an NNEA value of ~10^−10^ W cm^−1^ Hz^−1/2^ was obtained in this configuration. Recently, Zhang et al. [93] proposed a novel hydrogen sulfide detection system based on a doubly resonant QEPAS sensor with a line-locking mechanism. The authors introduced a doubly resonant system for both optical and acoustic fields to enhance sensitivity, and reached an NNEA of 8.9 × 10^−12^ W cm^−1^ Hz^−1/2^.

The QEPAS technique, while popular, is highly vulnerable to electromagnetic interference and necessitates a particular electrical read-out circuit, requiring a low-noise trans-impedance amplifier placed as close to the QTF as is practical.

#### 3.2.2. All-Optical Transducers

Although electromechanical transducers have good sensitivity and are quite mature technologically, they are not suitable for operation in severe environments since they heavily rely on electrical circuits for signal filtering and amplification. On the other hand, optical transduction techniques are resistant to electromagnetic interference and can function remotely in harsh conditions, including high temperatures and corrosive or explosive settings. In most cases, they have higher sensitivity than electromechanical sensors. Among the different types of all-optical sensors, interferometric systems are most commonly used for PAS gas detection, as they detect small changes in the optical path caused by the acoustic signal. Different interferometric systems such as the Michelson interferometer (MI) and the Fabry–Perot interferometer (FPI) have been utilized for PAS-based gas detection. Most of these transduction methods are based on the optical read-out of a cantilever and are referred to as cantilever-enhanced PAS (CEPAS) sensors.

**Figure 3:**
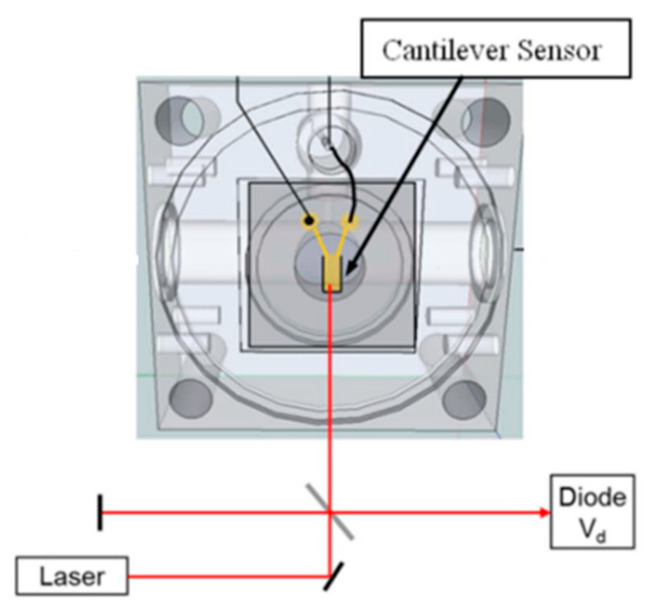
Schematic diagram of PA cell or chamber with cantilever sensor position [95].

Michelson interferometer—In the MI, the light is separated with a beam splitter and re-combines after reflection by two mirrors. The constructive or destructive interference condition depends on the position of one mirror, while the other stays still. The moving mirror is often a reflective cantilever with a free end, which has enhanced displacement. Figure 3 provides a general PAS gas detection system schematic based on this interferometric technique.

In this MI setup, the PA chamber and optics are mounted on an optical bench. A laser beam, guided through a series of mirrors, a beam splitter, irises, an attenuator, and focusing lenses, is reflected off the tip of the cantilever and back to a photodiode where the laser beam power is measured, providing the photoacoustic signal generated in the PA cell. This detection technique has been successfully employed by Laurila et al. [96], Fonsen et al. [97], Moser and Lendl [98], Kuusela et al. [99], Hirschmann et al. [100], McNaghten et al. [101], Cheng et al. [102], and many others and used for PAS gas detection with an NNEA of about 10^−10^ W cm^−1^ Hz^−1/2^.

The MI in CEPAS has many advantages over other all-optical PAS sensors, such as high sensitivity, excellent repeatability, and a sizeable dynamic range. However, it makes the system more complex, bulky, fragile, and vulnerable to environmental vibrations.

**Figure 4:**
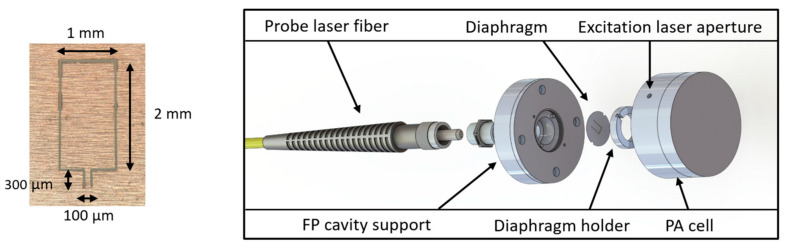
Schematic diagram of the cantilever microphone [107].

Fabry–Perot interferometer—The limitations of the MI can be addressed by the FPI. In the Fabry–Perot (FP) cavity, the displacement of one mirror also induces a phase shift, which causes a change in the resonance condition of the cavity. Based on an extrinsic FPI (EFPI), a recent transduction method for PAS consists of a reflecting mirror that is responsive to acoustic waves, which is typically the diaphragm, and a low-reflectivity interface, which is typically the cleaved end of a fiber. As a result, a cavity is formed between the diaphragm and the tip of the fiber. This approach offers the advantage of having great acoustic sensitivity while allowing simple alignment. As a result, though, the sensitivities are generally marginally lower than those obtained using the MI technique. The sensing mirror of the EFPI can be a simple circular diaphragm, such as silver [103], Parylene-C [104], graphene [105], titanium [106], stainless steel [107], silicon [108], and gold-coated polyetherimide (PEI) [109] diaphragms, all of which offer high reflectivity. Generally, the EFPI-based PAS technique is conducted according to the CEPAS method. The general structure of an FP cantilever optical microphone is given in Figure 4. The integrated sensor is composed of an optical fiber, a ceramic ferrule, a copper shell, and a cantilever, which constitute an FP cavity.

The typical NNEA obtained with the EFPI method lies in the 10^−7^–10^−9^ W cm^−1^ Hz^−1/2^ range. Compared with the other methods, this system shows greater compactness in structure, higher sensitivity, and greater suitability for optical-fiber-based remote sensing.

Numerous studies have been performed on developing cantilever-based EFPI sensors. Li et al. [110] developed an EFPI sensor with a stainless-steel diaphragm, and an NNEA coefficient as low as 2.1 × 10^−8^ W cm^−1^ Hz^−1/2^ was achieved, with an MDL of 8.4 ppm of CH_4_ with a 1 s integration time. This sensor structure can also be used in a dual-microphone-based gas detection system, and the detection limit can be further improved. Fu et al. [108] developed a dual-silicon-cantilever-microphone-based PAS system and estimated the NNEA coefficient to be 1.2 × 10^−9^ W cm^−1^ Hz^−1/2^ and the MDL for CH_4_ to be about 111.2 ppb with a 1 s integration time. White light interferometry is another technique through which sensitivity can be increased. Chen et al. [111] developed a white-light-interferometry-based all-optical PA spectrometer with a fiber optic FP cantilever microphone and achieved an NNEA coefficient of about 1.1 × 10^−9^ W cm^−1^ Hz^−1/2^. The same interferometry technique was also further developed for multi-gas detection, and the noise equivalent (minimum) detection limits achieved with a 100 s averaging time were 37, 9, 6, 17, 4, and 60 ppb for CH_4_, C_2_H_2_, C_2_H_4_, C_2_H_6_, CO, and CO_2_, respectively [112]. The cantilever could also be fabricated with a hinge, which can increase the sensitivity of the sensor. Lauwers et al. [107] developed a hinged-cantilever-based EFPI sensor and detected NO in N_2_ with an NNEA of 5.1 × 10^−7^ W cm^−1^ Hz^−1/2^. Guo et al. [113] developed a wavelength modulation spectroscopy (WMS) technique with a silicon-cantilever-fiber-optic-FPI-based PAS detection system and achieved a detection limit of 199.8 ppt of C_2_H_2_ with an averaging time of 60 s, and the NNEA was determined to be 1.72 × 10^−9^ W cm^−1^ Hz^−1/2^. The results indicate that better resolution could be achieved through WMS.

**Figure 5:**
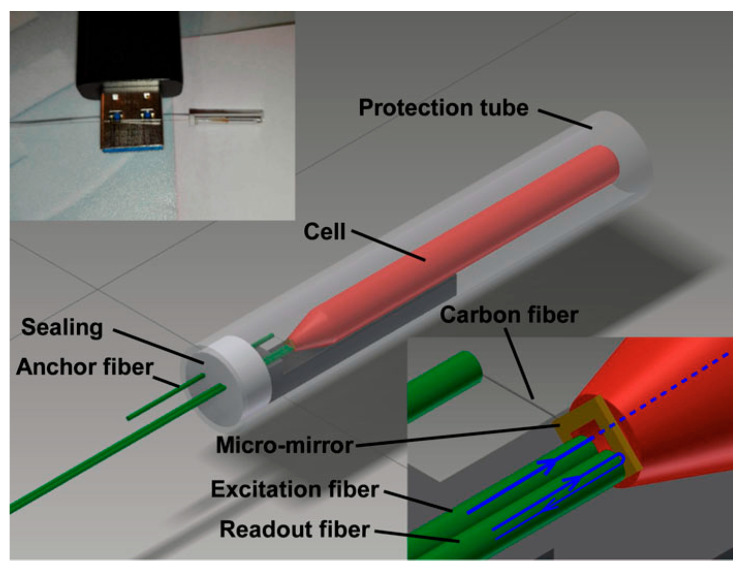
Sketch of the PAS sensor with a cantilever at the fiber tip [114].

Although less common, FP cavities can be directly fabricated at the tip of a fiber using additive microfabrication techniques. While this fabrication technique is complicated, the corresponding sensor offers high sensitivity by reducing viscous drag losses that traditional cantilevers experience. Zhou and Iannuzzi [114] and Zhou et al. [115] developed a miniaturized fiber-tip PAS sensor for in situ trace gas detection (Figure 5). In this method, the gas to be examined is contained in a tiny PA cell and excited with a modulated laser beam applied to the PA cell via an optical fiber. The acoustic wave from the PA excitation strikes a micromirror that is fastened to the free-hanging end of a cantilever beam. This spectrometer achieved a noise equivalent detection limit of 15 ppb for acetylene (C_2_H_2_) using a 23-mW excitation laser source, corresponding to an NNEA as low as 7.7 × 10^−10^ W cm^−1^ Hz^−1/2^. The stability of the quadrature operating point or Q-point is among the most important issues in EFPI. The Q-point is the operating point of the modulation also known as the bias point where the sensor exhibits the most sensitivity to the measurand and has a linear response. The Q-point drifts with the ambient temperature and if the probe laser’s wavelength doesn’t track the Q-point in real time, the demodulated PA signal will be incorrect. In addition, the limited linear functioning range at the Q-point limits the dynamic range.

### 3.3. Artificial Intelligence (AI)

Today, AI is used across various engineering fields to address issues more efficiently and automate many processes. Due to its potential for accurate and real-time processing, AI is being utilized in conjunction with PAS gas sensors to enhance sensitivity and selectivity by processing and filtering real-time signals from sensors simultaneously [116]. Studies have shown that machine learning algorithms are very effective in analyzing sensor data to accurately detect and distinguish different gases [117,118]. Tools such as wavelet analysis, neural networks, and sensor arrays are commonly used in machine learning [117,119].

To illustrate the corresponding applications, Lukić et al. [120] used a generalized regression neural network (GRNN) to estimate the PA signal parameters generated in methane (C_2_H_4_) and Ar gas mixture. Kozmin et al. [117] developed a new method that combines wavelet analysis and advanced neural network architectures to enhance the precision and sensitivity of PAS gas sensors. They evaluated the system using C_2_H_4_, and the results showed substantial advancements in gas detection technology. Wang et al. [57] developed a highly sensitive PAS system to detect C_2_H_2_ at ppb-level concentrations. This system uses a unique trapezoid compound ellipsoid resonant PA cell (TCER-PAC) and a partial least squares (PLS) regression algorithm. Their research revealed that the proposed PAS system, incorporating a TCER-PAC and the PLS algorithm, exhibited enhanced detection sensitivity and a 60-times lower detection limit than the PAS system without the regression algorithm. Li et al. [118] used AI to enhance the CO detection sensitivity of the PAS technique. They investigated the use of an empirical mode decomposition (EMD) algorithm for processing, especially in terms of nonlinear signal processing and noise compensation.

Moreover, optimization algorithms are more popular ways of integrating computational intelligence. As an example, Lukić et al. [121] explored computational intelligence and its use in pulse PAS. They merged particle swarm optimization and artificial bee colony optimization to enhance the precision of trace gas measurements. Additionally, they also examined the application of neural networks [122]. Lukić et al. [123] utilized a genetic algorithm for PAS signal temporal shape analysis and energy density spatial distribution calculations. Various optimization methods have been employed in PA analysis for various purposes, including calibration procedures [124], the simultaneous determination of PA signal parameters [125], the determination of trace gas concentrations, and the enhancement of the characteristics of PA cells.

## 4. Applications of PAS Gas Sensors

As they allow the quantitative detection of gases, PAS-based gas sensors have a wide range of applications, such as pollution monitoring, toxic gas detection, non-invasive medical diagnostics, and industrial process control, that require the detection of gases at ppb or sub-ppb levels [27,68]. These sensors can be utilized to detect gases such as ozone, ammonia, carbon dioxide, nitric oxide, and hexamethyldisilazane for environmental and medical purposes. Some of the main applications are discussed below.

Environmental Monitoring: In recent years, PAS gas sensors have been developed to identify various trace gases, and their applications are rapidly increasing in the field of atmospheric environmental gas monitoring, enabling the identification of gases and the quantitative measurement of concentrations to control and prevent air pollution. For example, Si et al. [126] reported a PAS gas detection method in which a near concentric cavity was used to detect NO_2_ at a level of 10.15 ppb, while Chen et al. [66] detected SO_2_ at a level of 140 ppb by designing a differential two-resonator PA cell. Similarly, Wang et al. [127] developed a dual-differential PA cell with four microphones to detect the greenhouse gases CH_4_ and CO_2_ at levels of 42 ppb and 0.86 ppb, respectively.

Regarding real-world applications, D’Urso et al. [128] utilized an infrared PA multi-gas analyzer to monitor NH_3_, CH_4,_ and CO_2_ concentrations in an open dairy barn. Liu et al. [129] used a PAS gas sensor for multiple-greenhouse-gas detection in the Qinling mountainous region of China. Dunker et al. [130] discussed the potential of utilizing PAS gas sensors for wearable benzene detection sensors. Furthermore, Li et al. [131] developed a fiber optic PAS gas sensing system for the multi-point remote monitoring of gases.

Industrial Applications: Gas sensors are extensively utilized in industrial settings for leak detection, process monitoring, and safety assurance. Historical incidents such as the Hindenburg fire in 1937 and the explosion at the Fukushima power plant in 2011 emphasize the catastrophic potential of any concentration changes or leakages [132]. Industries such as petroleum processing, petrochemical production, oil and fat hydrogenation, fertilizer production, the metallurgical application sector, and electronics are especially sensitive to gas-related incidents, making gas sensors crucial for monitoring airflow and preventing disasters. In the chip-manufacturing industry, gas sensors are essential due to the necessity of high-purity gases for technology development and quality upgrades in the LED industry [133]. Additionally, gas sensors play a critical role in the food industry by ensuring food quality and safety at various stages such as production, processing, storage, and transportation [134]. This helps in monitoring freshness, preventing the consumption of spoiled products, and reducing the risk of foodborne illnesses [135].

For instance, Chen et al. [136] developed a PAS sensor for the real-time detection of dissolved gases in oil. Nebiker and Pleisch [137] combined PAS gas sensors with fire detection systems to monitor carbon dioxide levels for early fire warnings. Pushkarsky et al. [138] described using a CO_2_ laser-based PAS sensor for detecting ammonia at sub-ppb levels in semiconductor clean room environments. Kaur et al. [139] highlighted the advantages of PAS gas sensors in detecting ethylene in fruit supply chains compared to other gas sensors. Wu et al. [140] developed a single-fiber, double-cavity-enhanced PAS gas sensor for methane gas detection in coal mine environments to improve safety.

Biomedical Applications: Gas sensors play a crucial role in medical applications and have shown promising results in various areas such as medical diagnostics, monitoring therapy response, and drug delivery monitoring, particularly through breath analysis. For instance, Nidheesh et al. [141] developed a PA breath analysis sensor for diagnosing lung diseases, while Luo et al. [142] developed a similar sensor for mouth alcohol testing. As breath analysis provides a non-invasive method for diagnosing human health, Zheng et al. [143] integrated PAS sensors for the real-time and pre-diagnosis detection of kidney disease by analyzing ammonia in exhaled breath. Additionally, Lay-Ekuakille et al. [144] designed a portable PA multi-gas monitor for biomedical gas that can be used in applications like monitoring anesthetic gases in surgical environments.

## 5. Challenges and Limitations

While PAS gas sensors represent a promising method for selective gas detection and quantification of changes in gas concentrations, they come with several limitations and encounter various technical challenges that can impact their performance and practicality. These challenges are interconnected and can influence each other. The main technical challenge is achieving high sensitivity and selectivity to detect low concentrations of gases and differentiate between different gas types [14]. While current advancements in PAS gas sensors can achieve sensitivity in the ppb and ppt ranges for certain gases, there are still difficulties in achieving this level of sensitivity for all gases. Sensor design and modification efforts are being made to improve sensitivity. For instance, to remain selective while simultaneously detecting multiple gases, non-overlapping absorption lines will be necessary. This might result in choosing absorption lines with low absorption coefficient values; therefore, enhancing the absorption of the laser light by the gas molecules to generate detectable acoustic pressures will be necessary. One primary method is increasing the power of the excitation light source or incorporating an optical cavity in the PA cell. Nevertheless, the use of high-power laser sources is limited due to their high cost, component size, power consumption, and the need for laser collection and -processing devices [126]. Additionally, employing high-power laser sources can elevate the temperature in the PA cell and potentially lead to flammability if proper temperature management is not employed.

Sensitivity can also be improved by optimizing the PA cell structure. However, there are limitations to the internal diameter of the PA cell due to the laser beam width. The internal diameter needs to be greater than the width of the laser beam. Although improving beam quality can address this issue to a certain extent, there is still significant sidewall noise, which poses challenges for ultra-sensitive gas detection [145]. Additionally, the PA signal is linked to the concentration of gas, and the corresponding intensity variation is a weak signal that is difficult to distinguish [146].

The relaxation of the excited state generated by light absorption is not always instantaneous, especially when the light source is modulated in the kHz range. This can result in molecular relaxation occurring only at the nanosecond scale or shorter rather than quasi-instantaneously. Additionally, light absorption can lead to the excitation of molecules into long-lived excited states, and vibrational–vibrational interaction can result in delayed relaxation processes and the generation of delayed PA signals [147]. Several techniques have been proposed to address this issue. The frequency limit can be set by reducing the resonance frequency of a sensor by changing its dimensions [92]. The signal can also be reduced or increased depending on the gas matrix considered, which reduces sensor sensitivity. Recently, a couple of approaches have been developed to address the matrix effects in PAS [148]. One is based on statistical regression analyses such as the multilinear regression (MLR) [149,150] and partial least-squares regression (PLSR) [151,152] methods. The other is based on a digital twin (DT) of a gas detection system for monitoring methane in environments with complex gas compositions [153].

The portability of a sensor limits its real-world applications. To overcome this limitation, miniaturizing PAS sensors is crucial. However, maintaining sensor performance while integrating components such as lasers and microphones in a compact format poses a significant challenge [14]. Physical constraints such as those relating to fabricating the PA cell; matching the modulation frequency of the excitation laser with the cell’s acoustic resonance frequency, which must remain slower than the collisional relaxation process of the gas molecules; and matching the acoustic sensor resonance frequency hinder the optimization of the size of the PAS sensor [154]. Additionally, as sensors become smaller, the validity of the continuum hypothesis may no longer apply in modeling, making this process more complex and challenging. At a certain point, boundary conditions like velocity slip and temperature jump must be considered instead of continuous boundary conditions [154].

The operating principle of PAS is based on the acoustic signal it produces and is highly susceptible to surrounding noise and vibrations. As a result, PAS gas sensors often face difficulties in more demanding environments where mechanical vibrations are present, such as industrial settings, moving vehicles, and agricultural surroundings [155]. Moreover, the components of PAS gas sensors are predominantly made of metal, making them susceptible to acidic environments and limiting their durability in such conditions. The background signal, produced by the laser light absorption at the windows or walls of the PA cell, is also a significant factor in the MDL of the PAS sensor. Due to the matching frequency of the background signal and the PA signal generated by absorption in the gaseous phase, it is extremely challenging to eliminate the background signal through data analysis [147].

Photoacoustic spectroscopy gas sensors are equipped with gas inlets and outlets through which gas flows while gas properties are quantitatively measured. However, like other spectroscopic techniques, the detection rate is constrained by the speed with which gas flows through the PA gas cell, limiting the rapid detection of gas properties. Moreover, the necessary gas volume depends on the size of the PA cell, posing challenges such as the need to accommodate large gas intake volumes.

In real-world applications of PAS, it is frequently observed that a signal is produced not just by the substance being analyzed but also by other elements. In the mid-infrared wavelength spectrum, the presence of water vapor often leads to significant spectral interference due to its powerful absorption across almost the entire wavelength range [147]. Additionally, the PA cell was initially designed to identify a single type of gas, with the PA cell geometry and light source being selected accordingly. As a result, it has limited capability to detect multiple gas components simultaneously and is challenging to enhance under hardware constraints [156]. Although using light sources with varying wavelengths in a single PA cell can help one detect different gases, this approach is still constrained by the high cost of light sources and the time required to detect multiple gases.

## 6. Future Directions and Opportunities

In recent years, researchers have significantly advanced PAS sensors, ushering in a new era of improved performance and sensitivity. The current focus of PAS gas-sensing research involves the development of new sensor designs and utilizing advanced technologies to enhance microphone sensitivity, detection limits, selectivity, and response time. Key areas of investigation include the advancement of techniques and tools such as MEMS microphones [157], multi-pass retroreflection enhancement [65], PA cell geometry optimization, micro-embedded acoustic resonators [158], machine learning algorithms [117], and digital lock-in amplification [159]. Additionally, attention has been directed towards the use of high-power diode lasers [17], quantum cascade lasers [160], and fiber optic techniques [69] to further improve the detection limits of PAS. Additionally, ongoing research is focusing on miniaturizing PAS sensors to enhance their portability and utility [69], as well as consolidating their components for greater efficiency.

However, there is a need for further development of PAS to achieve its maximum potential. Future advancements in PAS gas sensors could concentrate on several key areas. These include multi-gas detection and miniaturization to the millimeter scale, enhancing sensitivity to the ppt level, and exploring various types of resonant PA cells, such as Helmholtz and T-type cells, to improve sensitivity and selectivity. Additionally, there is a focus on cost-effective gas leakage detection, integrating the Internet of Things (IoT) with PAS to broaden applications in smarter ways and reduce operational and maintenance costs. These advancements improve sensitivity, decrease response times, enhance detection limits, and mitigate external interferences, making PAS gas sensors more efficient and reliable for various applications such as environmental protection, medical monitoring, and industrial process control.

## 7. Conclusions

In conclusion, this literature review has synthesized and highlighted the key findings related to PAS gas sensors, providing a comprehensive understanding of their capabilities and limitations, and shedding light on potential future advancements and opportunities for development. This review shows significant implications of the PAS technique for future research and practical applications, paving the way for advancements in environmental monitoring, industrial safety, and medical diagnostics, thereby contributing to the future development of more effective and reliable PAS gas sensors for use in diverse real-world settings. This will subsequently lead to broader implications for public health, environmental sustainability, and industrial processes. 

## Data Availability

No new data were created or analyzed in this study.
